# Investigation of factors related to treatment planning of x‐ray SBRT and scanning carbon‐ion radiation therapy for early‐stage lung cancer patients

**DOI:** 10.1002/acm2.14618

**Published:** 2025-02-11

**Authors:** Yuya Miyasaka, Sung Hyun Lee, Hikaru Souda, Hongbo Chai, Miyu Ishizawa, Takuya Ono, Takashi Ono, Hiraku Sato, Takeo Iwai

**Affiliations:** ^1^ Department of Heavy Particle Medical Science Yamagata University Graduate School of Medical Science Yamagata Japan; ^2^ Department of Radiology Yamagata University Faculty of Medicine Yamagata Japan

**Keywords:** carbon ion radiation therapy, lung cancer, SBRT, treatment planning, VMAT

## Abstract

This study aimed to compare the treatment plans of x‐ray SBRT and scanning carbon ion radiation therapy (CIRT) for localized lung tumors, and to evaluate the dose dependence of tumor size tumor‐to‐heart distance. For phantom verification, we used a chest phantom with a spherical simulated tumor. Treatment plans for 3‐dimensional conformal radiation therapy (3D‐CRT), volumetric modulated arc therapy (VMAT), and CIRT were created. GTVs were created in sizes ranging from 0.5 to 5 cm in diameter, and the dependence of the lung dose on GTV diameter was evaluated for each treatment plan. For patient validation, 30 cases of localized lung tumors were analyzed. 3D‐CRT, VMAT, and CIRT treatment plans were developed, and DVH parameters were evaluated for each GTV size and GTV‐to‐heart distance. In both phantom and patient validations, the OAR doses were the lowest for CIRT. The lung dose increased with increasing GTV diameter for all three treatment plans. CIRT had the smallest ratio of lung dose increase to GTV diameter increase among the three treatment plans. Heart dose in CIRT was independent of GTV size and GTV‐to‐heart distance Conclusions: The results of the present study suggested that the use of scanning CIRT can reduce the OAR dose while guaranteeing the tumor dose compared to x‐ray SBRT. In addition, it was suggested that CIRT can treat patients with large GTV sizes while maintaining low lung and heart dose.

## 1 INTRODUCTION

Radiotherapy has an important role in treating localized lung cancer or metastatic lung tumors. Subsequent to the development of stereotactic body radiotherapy (SBRT) in x‐ray therapy, good outcomes have been reported,^1^, ^2^, ^3^, ^4^, ^5^, ^6^, ^7^, ^8^, ^9^ with some comparable to those of surgery.^10^, ^11^, ^12^ In contrast, carbon‐ion radiotherapy (CIRT) is now available for treating localized lung tumors. Because of the Bragg peak and sharp lateral penumbra of carbon‐ion beams,^13^ CIRT is highly effective for treating lung tumors, and many good results have been reported.^14^, ^15^, ^16^, ^17^, ^18^, ^19^ Recently, Miyasaka et al. summarized the outcomes of x‐ray SBRT and CIRT. They reported significantly better local control by CIRT than by x‐ray SBRT.^20^ The advantage of CIRT for lung tumor treatment is that it reduces the organ at risk (OAR) dose. Yoshida et al. reported that the OAR dose was significantly lower for CIRT than for x‐ray SBRT.^21^ Anderle et al. similarly reported a reduction in the OAR dose in CIRT.^22^ Thus, CIRT is considered a promising option for treating localized lung tumors, offering both local control equal to or better and a lower OAR dose than those of SBRT.

Tumor size is an important factor when considering the outcomes and toxicity in the treatment of lung tumors. Allibhai et al. reported that lung tumor size was associated with local control and grade ≥2 lung pneumonia.^23^ Similarly, Parker et al. reported that lung tumor gross tumor volume (GTV) size was associated with local control.^24^ Ebara et al. used passive scattering CIRT to evaluate the relationship between tumor size and dose‐volume histogram (DVH) parameters in three‐dimensional conformal radiotherapy (3D‐CRT) SBRT and CIRT.^25^ They noted that the lung dose increased with increasing planning target volume (PTV) size and concluded that CIRT has the advantage of a lower‐dose OAR than that of 3D‐CRT SBRT, especially for larger tumor volumes. Thus, it is clear that tumor size is an important factor in the selection of treatment modalities for lung tumors.

Irradiation technology continues to develop for both SBRT and CIRT. The use of intensity‐modulated radiotherapy (IMRT) in SBRT is increasing, and its dose distribution characteristics differ significantly from those of 3D‐CRT. Many reports have supported the use of IMRT in SBRT for lung tumors because IMRT can maintain tumor doses comparable to those of 3D‐CRT while simultaneously reducing OAR doses.^26^, ^27^, ^28^ Regarding CIRT, the current common irradiation method is scanning irradiation, which can form a more highly concentrated dose distribution to the tumor than the passive‐scattering irradiation method used in the early 2010s.^29^, ^30^ However, there is a lack of discussion regarding the characteristics of each modality for localized lung tumors in the current treatment environment with improved irradiation techniques. This study aimed to determine if the dose distribution of x‐ray SBRT and CIRT for localized lung tumors is related to tumor size and other factors when using current treatment techniques.

## 2 MATERIALS AND METHODS

This study was conducted in two phases: phantom and patient validation.

### 2.1 Phantom validation

**FIGURE 1:**
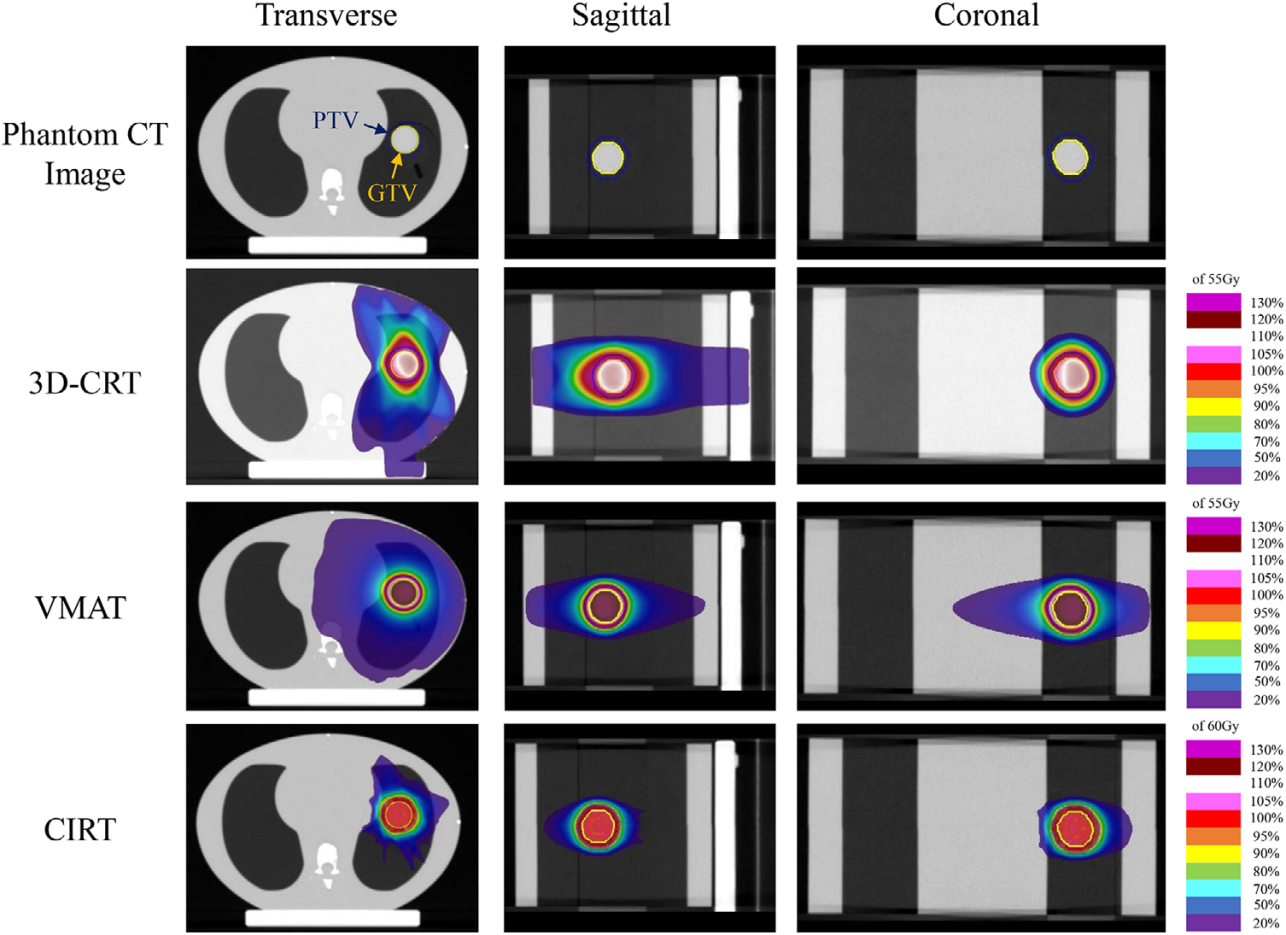
CT images of the phantom and dose distributions for the three treatment plans. Yellow ROIs indicate GTV and blue ROIs indicate PTV. 3D‐CRT, 3‐dimensional conformal radiation therapy; CI, conformity index; CIRT, carbon ion radiotherapy; *D*_max_, maximum dose; *D*_mean_, mean dose; *D*_x%_, minimum dose to the most irradiated x% of tissue volume; GTV, gross tumor volume; HI, homogeneity index; PTV, planning target volume; VMAT, volumetric modulated arc therapy; *V*_x%_, the ratio of the volume irradiated by x% or more of the prescribed dose; V_xGy_, the ratio of volume irradiated with xGy or more.

A respiratory synchronization phantom (CIRS Inc., Norfolk, USA) simulating the thoracic region was used for validation. Computed tomography (CT) images of the phantom were acquired on an Aquilion One (Canon Medical Systems, Otawara, Japan). A 2‐mm CT slice thickness was used at a voltage and current of 120 kV and 230 mA, respectively. Figure 1 shows the CT image of the phantom. The phantom was stationary and included 1‐, 2‐, and 3‐cm‐diameter inserted spheres for CT image acquisition. Each inserted sphere was designated as a test GTV. In addition, 0.5‐, 1.5‐, 2.5‐, 3.5‐, 4‐, 4.5‐, and 5.0‐cm‐diameter spherical regions of interest (ROIs) were created, and the ROI density was replaced by that of water (physical density; 1.0 g/cm^3^), which was then used as the GTV. This method allowed simulation of GTVs with diameters ranging from 0.5‐ to 5‐cm at 0.5‐cm intervals. The PTV was the GTV plus a 5‐mm margin. Then, three treatment plans were created for each size GTV from 0.5 to 5 cm: 3D‐CRT, volumetric modulated‐arc therapy (VMAT), and CIRT. A RayStation10A (RaySearch Laboratories, Stockholm, Sweden) was used to create all treatment plans. The dose‐calculation grid was 2 mm^3^ for all treatment plans. The 3D‐CRT and VMAT treatment plans were based on Versa HD (Elekta AB, Stockholm, Sweden) beam data with 6‐MV x‐rays. The dose‐calculation algorithm for 3D‐CRT and VMAT was the collapsed‐cone algorithm. Ten fields, including non‐coplanar beams, were used to create the 3D‐CRT treatment plan. The gantry and couch angles are presented in Table 1. Generally, the multi‐leaf collimator margin was 5 mm, and the dose‐distribution coverage was checked and adjusted accordingly within a few mm. Dual‐partial arcs from 300° to 180° were used to create the VMAT treatment plan. The CIRT treatment plan was based on the beam data of a full‐energy scanning carbon‐beam therapy system,^31^ and the irradiation method was a pencil‐beam scanning irradiation method.^32^ The CIRT used a pencil‐beam dose‐calculation algorithm. The microdosimetric kinetic model was used to calculate the relative biological effectiveness dose.^33^, ^34^ Four beams were used to create the CIRT treatment plan,^35^ with beam angles of 0°, 60°, 90°, and 330°. For 3D‐CRT and VMAT, the prescription was 55 Gy in four fractions, and 95% of the PTV was covered by the prescribed dose. For CIRT, the prescribed dose was 60 Gy in four fractions, and the PTV median dose was normalized to be equal to the prescribed dose. The relationships between the calculated conformity index (CI), homogeneity index (HI), and GTV diameter were calculated. The CI and HI of PTV were calculated from the following equations.

(1) $$\mathrm{CI}=\frac{V_{95\%,\mathrm{External}}}{V_{\mathrm{PTV}}}$$

(2) $$\mathrm{HI}=\frac{D_{2\%,\mathrm{PTV}}}{D_{98\%,\mathrm{PTV}}}$$

**TABLE 1:** Default setting values for gantry angle and couch angle for 3D‐CRT.

Beam number	Gantry angle (°)	Couch angle (°)
1	180	0
2	225	330
3	135	330
4	30	330
5	330	330
6	330	30
7	30	30
8	135	30
9	225	30
10	0	0

In Equation (1), *V*_95%,External_ represents the 95% dose of the prescription dose volume within the dose‐calculation region, and *V*_PTV_ represents the PTV. In Equation (2), *D*_2%,PTV_ and *D*_98%,PTV_ represent the *D*_2%_ and *D*_98%_ of the PTV, respectively. The ROI was created assuming that the low CT values area in the phantom was the lung, and DVH parameters were evaluated. The *D*_mean_, *V*_5%_, *V*_20%_, and *V*_40%_ of the lung‐GTV (area where the GTV was removed from the lung ROI) were evaluated, and their relationship to the GTV diameter was then calculated. The dose converted to the equivalent dose in 2‐Gy fractions (EQD2) was used to evaluate the lung‐GTV dose. The EQD2 conversion formula is shown in Equation (3).

(3) $$\mathrm{EQD}2=nd\frac{\left(d+\frac{\alpha }{\beta }\right)}{\left(2+\frac{\alpha }{\beta }\right)}$$

In Equation (3), *n* is the number of fractions and *d* is the fractional dose. In this study, $\frac{\alpha }{\beta }$ was set to 3 when evaluating the lung‐GTV.

### 2.2 Patient validation

We analyzed 30 patients treated with x‐ray SBRT or CIRT for localized lung tumors between 2020 and 2022. Among the analyzed cases, 29 were patients with stage‐1 lung cancer and one was a patient with a single lung metastasis. There were 16 cases of right‐lung tumors and 14 of left‐lung tumors. The institutional review board of our institution approved this study. CT images were obtained on an Aquilion LB or Aquilion One (Canon Medical Systems, Otawara, Japan). The CT imaging conditions were as follows: slice thickness, 2 mm; tube voltage, 120 kV; and tube current, 300 mA. For each CT image, the radiation oncologist created ROIs of the GTV, lungs, heart, and spinal cord. Definition of GTV delineation follows the protocol in the RTOG clinical trial.^36^, ^37^, ^38^ All ROIs of the GTV in each phase delineated by four‐dimensional (4D) CT were projected to the treatment planning CT under free breathing conditions. The GTVs projected on the treatment planning CT were merged, and this was designated as the internal GTV (IGTV). The phase of the 4DCT used to create the IGTV was the phase in which the shift of the center of mass of the GTV was < 5 mm. PTV was defined as IGTV with an isotropic margin of 5 mm. Three treatment plans were created for each CT image, 3D‐CRT, VMAT, and CIRT. All treatment plans were created on a RayStation10A. In principle, the treatment planning parameters for each treatment planning method were the same as those for the phantom validation. Seven fields, including non‐coplanar beams, were used to create the 3D‐CRT treatment plans. The beam angle was set to the angle listed in Table 1 as the default setting, and the angle was adjusted according to the tumor's location and size. The VMAT treatment plan was created in dual‐partial arcs according to the localization of the tumor. Four beams were used to create the CIRT plans. For CIRT plans, the number of beams was determined according to conventional clinical protocols.^18^, ^39^ The dose distribution of CIRT is sensitive to changes in particle range due to respiratory motion of the tumor, so special techniques are needed. Therefore, we used 4D robust optimization using 4DCT in CIRT treatment planning. 4D robust optimization allows treatment planning to account for changes in particle range due to respiratory motion.^40^, ^41^, ^42^

DVH parameters were evaluated for dose calculations performed on treatment‐planning CTs without CT value placement. In addition, we optimized for range uncertainty of 5% for robustness to range errors in treatment planning. The beam angle was set assuming a rotating gantry and was adjusted according to the tumor location. All treatment plans were optimized with the goal of achieving the constraints shown in Table 2. The *V*_98%_, *V*_95%_, *D*_2%_, CI, and HI were evaluated for the PTV. Equations (1) and (2) show the formulas for calculating CI and HI. Additionally, *R*_50%_ (ratio of the 50% prescription isodose volume to PTV) and *D*_2_ _cm_ (maximum dose at 2 cm from PTV) were calculated to evaluate dose falloff. For the OARs, Equation (3) was used to convert all dose distributions to EQD2, and the DVH parameters were evaluated. The value of α/β was set to 3. The *D*_mean_, *V*_5%_, *V*_20%_, and *V*_40%_ of the lung‐GTV and ipsilateral lung where the tumor was located, heart *D*_mean_ and spinal cord *D*_max_ were evaluated. The relationships between the IGTV diameter and the CI, HI, lung‐GTV *D*_mean_, *V*_5%_, V_20%_, and *V*_40%_ were calculated. The lung‐GTV DVH parameters were compared between the case groups with IGTV diameters larger and smaller than the median. For the heart dose, we evaluated the relationship between the IGTV diameter, GTV‐to‐heart distance, and *D*_mean_.

**TABLE 2:** Dose constraints.

Structure	Parameter	Constraint
PTV	*V*_95%_	>95%
Lung‐GTV	*D*_mean_	<18 Gy
	*V*_15Gy_	<25%
	*V*_20Gy_	<20%
Heart	*V*_30Gy_	<15 cc
Spinal cord	*D*_max_	<25 Gy

Abbreviations: *D*_max_, maximum dose; *D*_mean_, mean dose; PTV, planning target volume; *V*_x%_, the ratio of the volume irradiated by x% or more of the prescribed dose; *V*_xGy_, the ratio of volume irradiated with xGy or more.

### 2.3 Statistical analysis

The Statistical Package for the Social Sciences version 28 (IBM SPSS Statistics for Windows, IBM Corp., Armonk, NY, USA) was used for the statistical analysis. The Friedman and Bonferroni tests were used to evaluate significant differences because normality could not be confirmed by the Shapiro–Wilk tests in the comparison between the three treatment planning methods and in the respiratory motion evaluation. A *p*‐value of <0.05 was accepted as indicating statistical significance.

## 3 RESULTS

### 3.1 Phantom validation

**FIGURE 2:**
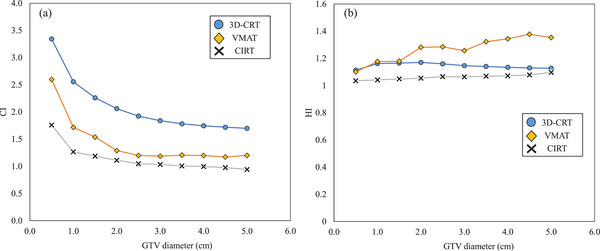
Relationship between the GTV diameter and the CI (a) and HI (b) in the treatment plan created on the CT image of the phantom. 3D‐CRT, 3‐dimensional conformal radiation therapy; CI, conformity index; CIRT, carbon ion radiotherapy; GTV, gross tumor volume; HI, homogeneity index; VMAT, volumetric modulated arc therapy.

**FIGURE 3:**
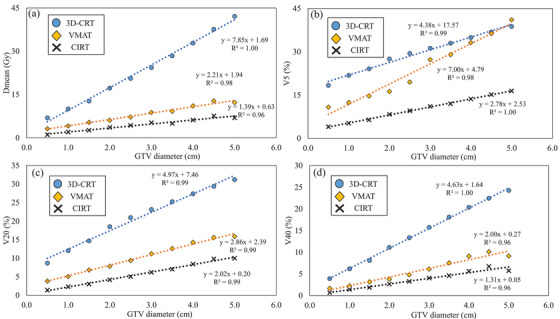
Relationship between GTV diameter and each DVH parameter of lung‐GTV on phantom CT images. *D*_mean_ is shown in (a), *V*_5_ _Gy_ in (b), *V*_20_ _Gy_ in (c), and *V*_40_ _Gy_ in (d). 3D‐CRT, 3‐dimensional conformal radiation therapy; CIRT, carbon ion radiotherapy; *D*_mean_, mean dose; GTV, gross tumor volume; VMAT, volumetric modulated arc therapy; *V*_xGy_, the ratio of volume irradiated with x Gy or more.

The relationships between the GTV size and CI and HI in the phantom validation are shown in Figure 2. In all three treatment plans, the CI approached 1.0 as GTV size increased. Among the three treatment plans, the CI for CIRT was closest to 1.0. The HI showed no dependence on GTV size for 3D‐CRT and CIRT but tended to be larger for VMAT as the tumor diameter increased. Figure 3 shows the relationship between the GTV size and DVH parameters of the lung‐GTV. All DVH parameters increased with increasing GTV diameter in all three treatment plans. All DVH parameters at the CIRT for all GTV sizes showed the smallest values among the three treatment plans. The slope of the approximate line for the CIRT was the lowest for all DVH parameters of the lung‐GTV. An example comparison of the three treatment plan dose distributions is shown in Figure 1. For the 3D‐CRT, the moderate‐to‐high 80%–70% dose range of the prescribed dose extended outside the PTV. The high‐dose region was narrower for the VMAT dose distribution than for the 3D‐CRT, but the low‐dose region of <20% of the prescribed dose was wider. In the CIRT dose distribution, the high‐ and low‐dose regions were located near the PTV, maintaining a low lung dose.

### 3.2 Patient validation

The median diameter of the GTV and IGTV were 2.6 cm (range: 1.2–4.5 cm) and 3.1 cm (1.2–4.9 cm) and the volume were 6.6 cc (range: 0.5–20.6 cc) and 9.0 cc (range: 0.7–25.2 cc). The average DVH parameters for all cases are listed in Table 3. For PTV, there was no significant difference between VMAT and CIRT at *V*_95%_. The *V*_98%_ was significantly lower in CIRT than in 3D‐CRT and VMAT. Among the three plans, the *D*_2%_ was significantly lower for CIRT. Among all treatment plans, the DVH parameters of all OARs were significantly lowest in CIRT. For the CI, significant differences were identified between 3D‐CRT and VMAT or CIRT, and no significant differences between VMAT and CIRT. The HI was 1.3 ± 0.2, 1.3 ± 0.1, and 1.0 ± 0.0 for the 3D‐CRT, VMAT, and CIRT, respectively. The *R*_50%_ of CIRT was significantly lower than that of 3D‐CRT and VMAT, and *D*_2_ _cm_ of CIRT was significantly lower only compared to 3D‐CRT.

**TABLE 3:** Average DVH parameter values for all cases.

		3D‐CRT	VMAT	CIRT
PTV	*V*_98%_ (%)	97.1 ± 1.6 (96.5–97.6)	97.9 ± 0.7 (97.6–98.1)	92.9 ± 5.2 (90.9–94.8)
	*V*_95%_ (%)	98.7 ± 1.0 (98.3–99.1)	99.2 ± 0.6 (99.0–99.5)	99.1 ± 1.5 (98.5–99.6)
	*D*_2%_ (%)	130.1 ± 14.2 (124.4–135.1)	129.0 ± 7.3 (126.5–132.0)	101.1 ± 0.4 (101.0–101.3)
	CI	1.6 ± 0.2 (1.5–1.6)	1.3 ± 0.2 (1.3–1.4)	1.3 ± 0.2 (1.2–1.3)
	HI	1.3 ± 0.2 (1.3–1.4)	1.3 ± 0.1 (1.3–1.4)	1.0 ± 0.0 (1.0–1.1)
	*R*_50%_	5.5 ± 0.7 (5.2–5.7)	5.6 ± 1.1(5.1–6.0)	4.1 ± 1.2 (3.7–4.6)
	*D*_2_ _cm_ (%)	70.7 ± 11.2 (66.5–75.0)	62.0 ± 6.1 (59.7–64.3)	59.1 ± 10.6 (55.1–63.1)
Lung‐GTV	*D*_mean_ (Gy)	11.0 ± 4.8 (9.2–12.8)	10.7 ± 4.5 (9.0–12.4)	7.9 ± 3.4 (6.5–9.2)
	*V*_5_ _Gy_ (%)	18.0 ± 7.1 (15.3–20.7)	18.7 ± 7.6 (15.8–21.6)	8.3 ± 3.2 (7.1–9.5)
	*V*_10_ _Gy_ (%)	14.7 ± 5.9 (12.4 ‐16.9)	13.1 ± 5.1 (11.1–15.0)	7.3 ± 2.9 (6.1–8.4)
	*V*_20_ _Gy_ (%)	11.0 ± 4.9 (9.2–12.9)	9.7 ± 4.0 (8.2–11.2)	6.0 ± 2.5 (5.0–6.9)
	*V*_30_ _Gy_ (%)	8.2 ± 4.0 (6.7–9.7)	7.6 ± 3.3 (6.4–8.9)	5.0 ± 2.1 (4.2–5.7)
	*V*_40_ _Gy_ (%)	6.0 ± 2.8 (4.9–7.0)	6.2 ± 2.8 (5.1–7.3)	4.2 ± 1.8 (3.5–4.9)
Ipsilateral lung	*D*_mean_ (Gy)	21.5 ± 10.7 (17.4–25.5)	19.6 ± 8.4 (16.5–22.8)	15.4 ± 7.2 (12.7–18.2)
	*V*_5_ _Gy_ (%)	33.2 ± 13.0 (28.2–38.1)	30.2 ± 10.6 (26.2–34.2)	15.9 ± 6.5 (13.4–18.3)
	*V*_10_ _Gy_ (%)	27.5 ± 11.4 (23.2–31.8)	24.2 ± 9.6 (20.6–27.9)	13.9 ± 5.9 (11.7–16.2)
	*V*_20_ _Gy_ (%)	21.1 ± 9.6 (17.4–24.7)	18.6 ± 8.0 (15.6–21.6)	11.5 ± 5.0 (9.6 ‐13.4)
	*V*_30_ _Gy_ (%)	15.8 ± 8.0 (12.8–18.8)	14.8 ± 6.6 (12.3‐ 17.3)	9.6 ± 4.2 (8.0–11.2)
	*V*_40_ _Gy_ (%)	11.8 ± 6.1 (9.5–14.1)	12.1 ± 5.6 (10.0–14.2)	8.3 ± 3.8 (6.9–9.8)
Heart	*D*_mean_ (Gy)	4.0 ± 5.8 (1.7–6.2)	4.6 ± 6.6 (2.1–7.2)	0.2 ± 0.3 (0.1 ‐0.3)
Spinal cord	D_max_ (Gy)	11.2 ± 14.2 (5.9–16.6)	15.9 ± 8.8 (12.6–19.3)	3.9 ± 9.4 (0.4–7.5)

		*p*‐value (3D‐CRT vs. VMAT)	*p*‐value (VMAT vs. CIRT)	*p*‐value (3D‐CRT vs. CIRT)
PTV	*V*_98%_ (%)	0.042*	<0.001*	<0.001*
	*V*_95%_ (%)	0.047*	0.454	0.682
	*D*_2%_ (%)	1.000	<0.001*	<0.001*
	CI	<0.001*	0.258	<0.001*
	HI	1.000	<0.001*	<0.001*
	*R*_50%_	1.000	<0.001*	<0.001*
	*D*_2_ _cm_ (%)	<0.001*	0.578	0.001*
Lung‐GTV	*D*_mean_ (Gy)	1.000	<0.001*	<0.001*
	*V*_5_ _Gy_ (%)	0.532	<0.001*	<0.001*
	*V*_10_ _Gy_ (%)	0.001*	<0.001*	<0.001*
	*V*_20_ _Gy_ (%)	0.001*	<.0001*	<0.001*
	*V*_30_ _Gy_ (%)	0.186	<0.001*	<0.001*
	*V*_40_ _Gy_ (%)	0.865	<0.001*	<0.001*
Ipsilateral lung	*D*_mean_ (Gy)	0.236	<0.001*	<0.001*
	*V*_5_ _Gy_ (%)	0.004*	<0.001*	<0.001*
	*V*_10_ _Gy_ (%)	<0.001*	<0.001*	<0.001*
	*V*_20_ _Gy_ (%)	0.002*	<0.001*	<0.001*
	*V*_30_ _Gy_ (%)	0.152	<0.001*	<0.001*
	*V*_40_ _Gy_ (%)	1.000	<0.001*	<0.001*
Heart	*D*_mean_ (Gy)	0.811	0.002*	0.004*
Spinal cord	*D*_max_ (Gy)	0.264	<0.001*	0.068

*Note*: Values are mean+1SD (95% confidence interval). Significant *p* values are indicated by *.

Abbreviations: 3D‐CRT, 3‐dimensional conformal radiation therapy; CI, conformity index; CIRT, carbon ion radiotherapy; *D*_max_, maximum dose; *D*_mean_, mean dose; *D*_x%_, minimum dose to the most irradiated x% of tissue volume; HI, homogeneity index; PTV, planning target volume; *R*_50,_ ratio of the 50% prescription isodose volume to PTV, *R*_2cm_, maximum dose at 2 cm from PTV; VMAT, volumetric modulated arc therapy; V_xGy_, the ratio of volume irradiated with xGy or more; *V*_x%_ = the ratio of the volume irradiated by x% or more of the prescribed dose.

**FIGURE 4:**
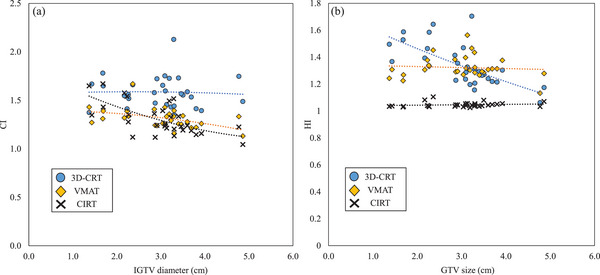
Relationship between IGTV diameter and CI (a) and HI (b) for three treatment plans created on patient CT images. 3D‐CRT, 3‐dimensional conformal radiation therapy; CI, conformity index; CIRT, carbon ion radiotherapy; GTV, gross tumor volume; HI, homogeneity index; VMAT, volumetric modulated arc therapy.

**FIGURE 5:**
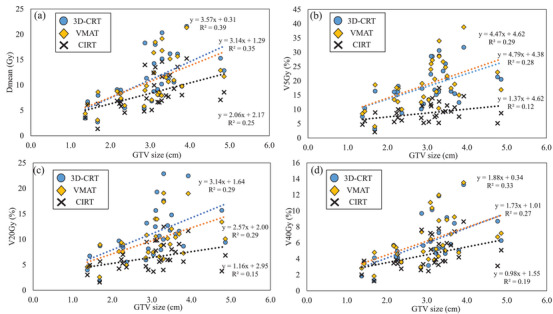
Relationship between IGTV diameter and DVH parameters of lung‐GTV in three treatment plans created on patient CT images. *D*_mean_ is shown in (a), *V*_5_ _Gy_ in (b), *V*_20_ _Gy_ in (c), and *V*_40 Gy_ in (d). 3D‐CRT, 3‐dimensional conformal radiation therapy; CIRT, carbon ion radiotherapy; *D*_mean_, mean dose; GTV, gross tumor volume; VMAT, volumetric modulated arc therapy; *V*_xGy_, the ratio of volume irradiated with x Gy or more.

The relationships between the IGTV diameter and CI and HI are shown in Figure 4. For the CI, all three treatment plans had values close to 1.0 as the GTV size increased. For the HI, there was IGTV diameter dependence in 3D‐CRT and no dependence in VMAT and CIRT. The relationship between IGTV diameter and the DVH parameters of the lung‐GTV is shown in Figure 5. The DVH parameter values for the lung‐GTV tended to increase as the IGTV diameter increased. The slope of the approximate line in CIRT was the lowest, followed by those of the VMAT and 3D‐CRT.

**FIGURE 6:**
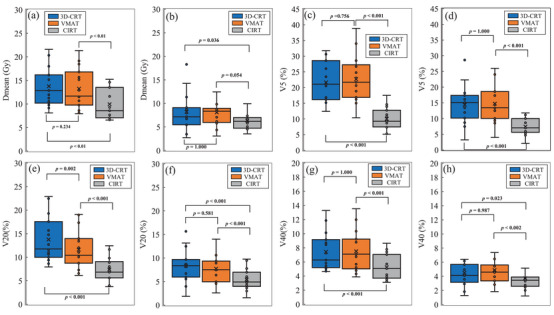
Box‐and‐whisker diagram of lung‐GTV for each case group with larger and smaller than median IGTV diameter (3.1 cm). *D*_mean,_ *V*_5_ _Gy_, *V*_20_ _Gy_, and *V*_40_ _Gy_ for the case group larger than the median IGTV diameter are (a), (c), (e), and (g), respectively, and *D*_mean_, *V*_5_ _Gy_, *V*_20_ _Gy_, and *V*_40_ _Gy_ for the case group smaller than the median GTV diameter are (b), (d), (f), and (h), respectively. 3D‐CRT, 3‐dimensional conformal radiation therapy; CIRT, carbon ion radiotherapy; *D*_mean_, mean dose; GTV, gross tumor volume; VMAT, volumetric modulated arc therapy; *V*_xGy_, the ratio of volume irradiated with x Gy or more.

A box‐and‐whisker plot of the IGTV diameter divided into groups with a IGTV diameter larger and smaller than the median (3.1 cm) is shown in Figure 6. There were significant differences between CIRT and 3D‐CRT or VMAT in all parameters except *D*_mean_. In contrast, there was a significant difference between 3DCRT and VMAT only in the *V*_20_ _Gy_ IGTV diameter larger group. For all DVH parameters, the group with larger‐IGTV diameters tended to have larger differences among the three treatment plans than the group with smaller IGTV diameters. For example, for the *D*_mean_, the mean differences between 3D‐CRT and VMAT, VMAT and CIRT, and 3DC‐CRT and CIRT, were 0.6 , 3.3, and 3.9 Gy, respectively, in the large‐GTV group, and were 0.1, 1.4 , and 1.5 Gy, respectively, in the small‐GTV group.

**FIGURE 7:**
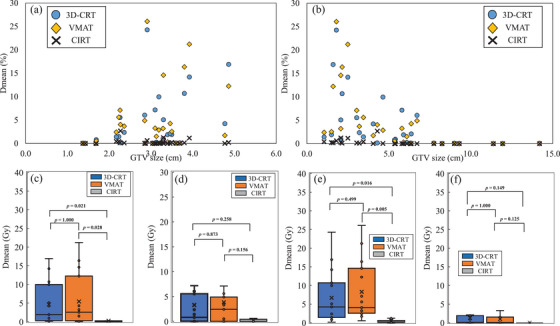
Relationship between IGTV diameter and GTV‐to‐heart distance and heart *D*_mean_. (a) and (b) are scatter plots showing the relationship between IGTV diameter and GTV‐to‐heart distance and heart *D*_mean_, respectively. Groups with IGTV diameter larger than the median IGTV diameter (3.1 cm) are shown in (c) and those with IGTV diameter smaller than the median IGTV diameter are shown in (d). Groups closer than the median GTV‐to‐heart distance (5.4 cm) are shown in (e), and groups farther away are shown in (f). 3D‐CRT, 3‐dimensional conformal radiation therapy; CIRT, carbon ion radiotherapy; *D*_mean_, mean dose; GTV, gross tumor volume; VMAT, volumetric modulated arc therapy.

Figure 7 shows scatter plots of the relationships between the heart *D*_mean_ and IGTV diameter (Figure 7a) and the GTV‐to‐heart distance (Figure 7b). For 3D‐CRT and VMAT, the heart dose tended to increase with increasing IGTV diameter, whereas for CIRT, there was little change in the heart dose. There was no significant difference in heart *D*_mean_ between 3D‐CRT and VMAT regardless of GTV diameter and GTV‐to‐heart distance. The difference between CIRT and 3D‐CRT or VMAT was larger for the larger IGTV diameter. For the GTV‐to‐heart distance and heart *D*_mean_, the differences between CIRT and 3DCRT and VMAT were greater in the group closer (Figure 7e) than the median of 5.4 cm than in the group that was farther (Figure 7f).

**FIGURE 8:**
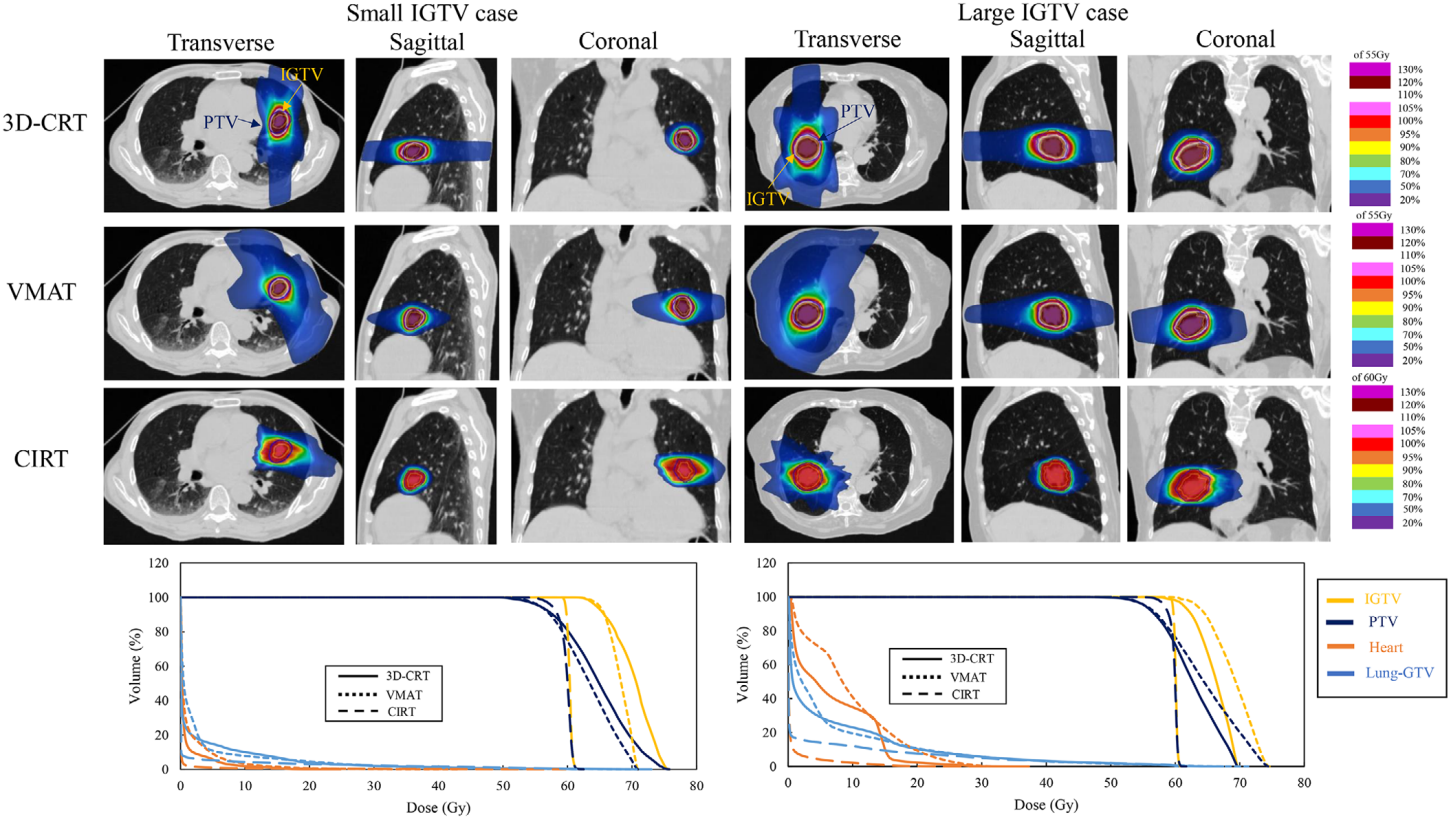
Dose distribution and DVH of three treatment plans for cases with smaller than median IGTV diameter and for cases with larger. 3D‐CRT, 3‐dimensional conformal radiation therapy; CIRT, carbon ion radiotherapy; DVH, dose volume histogram; GTV, gross tumor volume; PTV, planning target volume; VMAT, volumetric modulated arc therapy.

Figure 8 shows a typical example of the dose distribution and DVH. Compared with the small‐IGTV case, the large‐IGTV case had an extended high‐dose area in 3D‐CRT and an extended low‐dose area in VMAT. CIRT also showed a larger spread in the dose distribution for the large‐IGTV case, but the change was smaller than those of the other two treatment plans.

## 4 DISCUSSION

In this study, we compared the treatment plans of x‐ray SBRT and scanning‐beam CIRT in localized lung tumors and evaluated the dependence of the dose distribution on IGTV size and GTV‐to‐heart distance. As shown in Table 3, the OAR dose was significantly reduced for CIRT than for 3D‐CRT and VMAT. These results suggested that treatment with CIRT can be implemented with significantly reduced toxicity. Similar to these study results, those in previous studies indicated a reduced OAR dose when using CIRT than when using 3D‐CRT and VMAT.^21^, ^22^, ^43^ In the case of x‐ray SBRT, previous studies have reported that the OAR dose was lower for VMAT than for 3D‐CRT,^26^, ^27^ which was also observed in our study. However, the OAR dose was lower for CIRT even than for VMAT (Table 3). As shown in the dose distributions (Figures 1 and 8), the spread of high doses outside the PTV was smaller for VMAT than for 3D‐CRT, but the spread of low doses was comparable to that of 3D‐CRT. In contrast, our results suggest that CIRT can reduce the spread not only in the high‐dose area but also in low‐dose area. In addition, the *R*_50%_ and *D*_2_ _cm_ evaluations revealed that CIRT has a steeper dose falloff than 3DCRT and VMAT. This low spread in the high‐ and low‐dose areas is considered significant in actual clinical practice. For example, Ono et al. reported that the smaller dose spread of proton therapy than for x‐rays was safer in patients with idiopathic lung pulmonary fibrosis.^44^ In addition, Okano et al. reported that carbon ion therapy is a modality that can provide safe treatment for lung cancer patients with interstitial lung disease.^45^ Considering the results of this study, scanning CIRT, which resulted in sufficiently low OAR doses in our study, may be an effective option for the treatment of lung tumors in patients with comorbidities.

The phantom validation results showed that all treatment plans, 3D‐CRT, VMAT, and CIRT, showed linear increases in the lung dose with increasing GTV diameter (Figure 3). Of these, CIRT had the lowest slope of the linear line. This indicates the smallest increase in lung dose for an increase in tumor diameter. The fact that this trend was shown to be similar in this study suggests that in real patients (Figure 5), as in the phantom validation, CIRT should show a slow increase in lung dose in relation to tumor diameter. In addition, the difference in the DVH parameters for lung‐GTV in 3D‐CRT or VMAT and CIRT was greater in cases with larger tumor sizes, as shown when the groups were divided by their median IGTV diameters (Figure 6). This finding suggests that CIRT can be used to treat patients with large‐IGTV diameters with a reduced increase in the lung dose. Ebara et al. evaluated the relationship between lung dose and tumor volume for x‐ray SBRT and CIRT.^25^ They reported that the lung dose tended to increase with increasing PTV volume for both 3D‐CRT and CIRT, which was similar to the present study results. However, in their results, there were several parameters for which the slopes of the approximate straight line representing the relationship between the PTV volume and lung dose for 3D‐CRT and CIRT were similar, which differed from the results of our study. This may be due to the difference in the irradiation methods between their study and ours. In their study, the CIRT irradiation method was the passive‐scattering irradiation technique; however, in our study, we used a scanning irradiation technique. Several studies have reported that a more tumor‐limited dose distribution is obtained with the scanning irradiation technique than with the passive‐scattering irradiation technique.^29^, ^30^ For example, Shiomi et al. compared the dose distribution between passive‐ and scanning irradiation of CIRT in pancreatic cancer cases.^30^ In this report, they showed that the intestinal dose around the tumor may be significantly lower for scanning irradiation than for passive irradiation. The present study showed that the lung dose was reduced in CIRT using the scanning irradiation method relative to that reported by Ebara et al.^25^ resulting in a smaller ratio of lung dose increase to tumor size.

As shown in Table 3, the difference in mean *V*_95%_ was less than <0.6% between the three treatment plans, a small difference. This means that any of the three treatments can deliver a sufficient dose to control the tumor. In contrast, the *V*_98%_ was significantly lower in CIRT than in 3D‐CRT or VMAT. This finding may be due to differences in the normalization methods used for dose prescription. In this study, the doses were normalized so that the 100% dose covered 95% of the volume of the PTV for 3D‐CRT and VMAT, and the median dose was 100% of the PTV dose for CIRT. Reports using volume‐prescribing methods, such as the present study for x‐ray SBRT, instead of the reference point prescribing method have been increasing.^6^, ^46^, ^47^, ^48^, ^49^ On the basis of these reports, the volume prescription method was used in the present study. The standardized methodology of this study in CIRT follows our clinical protocol and is similar to that in previous publications showing clinical results.^20^ Although there is a difference in the normalization technique, the difference in dose prescription between x‐rays and particle beams has not been shown to affect tumor control, and the *V*_98%_ difference in this study cannot be attributed to tumor control. The significantly higher *D*_2%_ and HI for 3D‐CRT and VMAT than for CIRT may also be attributed to this difference in the normalization methods. Recently, the method of increasing the central dose and steepening the dose gradient has become popular in x‐ray SBRT.^47^, ^49^, ^50^ At this time, all of the normalization methods used in this study, 3D‐CRT, VMAT, and CIRT, were considered to be clinically used and to deliver acceptable tumor doses.

As shown in Table 3, the mean values of CI were 1.6 ± 0.3, 1.3 ± 0.2, and 1.3 ± 0.2 for 3D‐CRT, VAMT, and CIRT, respectively. In addition, as shown in Figure 4, the CI was closer to 1.0 in more cases in CIRT than in 3D‐CRT or VMAT. This finding indicates that CIRT can focus higher doses on the tumor, in contrast to 3D‐CRT and VMAT, and Ebara et al. stated the same conclusion for CI.^25^ Although the difference in CI between VMAT and CIRT was small, the CI was significantly higher for 3D‐CRT. This finding suggests that a higher dose can be delivered to the tumor by VMAT than by 3D‐CRT. However, as mentioned above, the key point to consider with VMAT is that low doses are distributed over a wider area. As shown in Figure 6, the low doses were spread out more widely in cases with larger‐IGTV diameters than with smaller GTVs. Therefore, our results indicate that CIRT has an advantage over VMAT because it can focus higher doses on the tumor and reduce lower doses.

In this study, the HI of 3D‐CRT was higher for smaller IGTV diameters (Figure 4). This is because the smaller diameter of the IGTV increases the proportion of low‐density lung area contained within the PTV, and also because the irradiation field is narrowed to emphasize dose concentration. The HI of VMAT tended to be higher with 3DCRT in cases with larger IGTV diameters. This may be due to the higher maximum dose (*D*_2%_) in the tumor while the target coverage is the same for 3DCRT and VMAT. The reason is that in this study we did not set a strong constraint on the maximum tumor dose in our VMAT optimization. In recent years, good outcomes have been reported in early‐stage lung cancer SBRT by increasing the maximum tumor dose.^50^, ^51^, ^52^ This would seem to justify to some extent the worsening of HI by increasing the maximum tumor dose.

For 3D‐CRT and VMAT, the heart doses depended on changes in the IGTV diameter and GTV‐to‐heart distance (Figure 7a and b). In contrast, there was little change in the heart dose with CIRT. A comparison of the median IGTV diameter and median GTV‐to‐heart distance between the two groups showed that the *D*_mean_ differences in CIRT, 3D‐CRT, and VMAT were larger in the group with a larger IGTV diameter size and in the group with a closer GTV‐to‐heart distance (Figure 7c–f). These findings suggest that the benefit of heart‐dose reduction by CIRT is greater in cases with larger IGTV diameters and closer‐GTV‐to‐heart distances. Previous reports have shown that heart doses can be significantly lower with CIRT than with x‐ray SBRT,^21^, ^25^, ^43^ but the factors involved have not been clarified. In the present study, since the mean dose for x‐ray SBRT increased significantly when the distance between the GTV and heart was <6.6 cm (Figure 7b), CIRT may have an advantage in terms of heart dose when the distance between the GTV and heart is <6.6 cm. Furthermore, since the IGTV size had an effect on heart dose, the GTV size and GTV‐to‐heart distance could serve as indicators of choice between x‐rays and CIRT.

A limitation of this study was the difference in the dose‐calculation algorithms. The collapsed‐cone algorithm was used for 3D‐CRT and VMAT, and the pencil‐beam algorithm was used for CIRT. Although there should be no problem in applying the results of this study in actual clinical practice given that both algorithms are used clinically, we have not been able to consider the effects of algorithm differences. Although the introduction of inhomogeneity‐correction algorithms in particle therapy has lagged behind that of x‐rays, highly accurate algorithms have become commercially available for particle therapy in recent years.^53^, ^54^, ^55^ In the future, the present study results can be evaluated in more detail by examining the algorithm in a unified environment. In radiotherapy of lung cancer cases, the robustness of the treatment plan to respiratory motion is especially important for particle therapy. In x‐ray treatment planning, the results of several papers suggest that irradiation at a respiratory motion of the tumor <5 mm, as assumed in this study, would have minimal impact on this study results.^56^, ^57^ In contrast, the importance of robust evaluation is considered to be higher in particle therapy than in x‐ray therapy. In the CIRT treatment plan, the difference in PTV *V*_95%_ calculated between the CT of each respiratory phase obtained from 4DCT and the treatment plan CT, respectively, was 1.4% ± 4.1% for all cases. This difference is not considered to significantly change the trend of the present results. Exhaustive 4D robust evaluation in all cases was not performed in this study. This is because in some cases the whole lungs and some OARs were not in the 4DCT imaging area. Further additional analysis of the results of this study for respiratory migration will be important in the future with a more comprehensive evaluation.

## 5 CONCLUSIONS

We evaluated and characterized the treatment plans for x‐ray SBRT and scanning CIRT for localized lung tumors. The study results suggest that scanning CIRT can be used to develop treatment plans with higher tumor dose conformity and lower OAR doses than those of x‐ray SBRT. The results suggest, especially in the case of large tumors that CIRT could minimize the increase in lung and heart dose while ensuring an adequate tumor dose. Furthermore, in cases in which the tumor and heart were in close proximity, CIRT was able to reduce the heart dose. Therefore, CIRT appears to be a particularly effective choice when the dose to the lungs and heart must be kept low.

## AUTHOR CONTRIBUTION

**Yuya Miyasaka**: Writing the manuscript; creating a treatment plan, data analysis, statistical analysis. **Sung Hyun Lee**: Data analysis and review manuscript. **Hikaru Souda**: Data analysis and review manuscript. **Hongbo Chai**: Review data and manuscript review. **Miyu Ishizawa**: Review data and review manuscript. **Takuya Ono**: Review data and manuscript review. **Takashi Ono**: Contouring ROIs; clinical integration; clinical review; review manuscript. **Hiraku Sato**: Clinical integration; clinical review; review manuscript. **Takeo Iwai**: Management and coordination responsibility for the research activity planning and execution.

## CONFLICT OF INTEREST STATEMENT

The authors declare no conflicts of interest.

## Data Availability

Research data are stored in an institutional repository and will be shared upon request to the corresponding author.
